# Fast and Quantitative T1ρ-weighted Dynamic Glucose Enhanced MRI

**DOI:** 10.1038/srep42093

**Published:** 2017-02-07

**Authors:** Patrick Schuenke, Daniel Paech, Christina Koehler, Johannes Windschuh, Peter Bachert, Mark E. Ladd, Heinz-Peter Schlemmer, Alexander Radbruch, Moritz Zaiss

**Affiliations:** 1 German Cancer Research Center (DKFZ), Division of Medical Physics in Radiology, Im Neuenheimer Feld 280, 69120 Heidelberg, Germany; 2 German Cancer Research Center (DKFZ), Division of Radiology, Im Neuenheimer Feld 280, 69120 Heidelberg, Germany; 3 High-field Magnetic Resonance Center, Max-Planck-Institute for Biological Cybernetics, Spemannstraße 41, 72076 Tübingen, Germany

## Abstract

Common medical imaging techniques usually employ contrast agents that are chemically labeled, e.g. with radioisotopes in the case of PET, iodine in the case of CT or paramagnetic metals in the case of MRI to visualize the heterogeneity of the tumor microenvironment. Recently, it was shown that natural unlabeled D-glucose can be used as a nontoxic biodegradable contrast agent in Chemical Exchange sensitive Spin-Lock (CESL) magnetic resonance imaging (MRI) to detect the glucose uptake and potentially the metabolism of tumors. As an important step to fulfill the clinical needs for practicability, reproducibility and imaging speed we present here a robust and quantitative T_1ρ_-weighted technique for dynamic glucose enhanced MRI (DGE-MRI) with a temporal resolution of less than 7 seconds. Applied to a brain tumor patient, the new technique provided a distinct DGE contrast between tumor and healthy brain tissue and showed the detailed dynamics of the glucose enhancement after intravenous injection. Development of this fast and quantitative DGE-MRI technique allows for a more detailed analysis of DGE correlations in the future and potentially enables non-invasive diagnosis, staging and monitoring of tumor response to therapy.

In diagnostic imaging it is often beneficial to enhance the contrast in tissue or to make a contrast more specific to a certain physiology or pathology. This is usually achieved by chemical labeling of specific agents, for example by labeling metabolites with radioisotopes in the case of PET, making use of iodinated compounds in the case of CT or using chelated paramagnetic metals in the case of MRI. As a paradigm shift, it was shown that natural unlabeled D-glucose could serve as a biodegradable contrast agent for the detection of cancer by employing chemical exchange saturation transfer (CEST) or chemical exchange sensitive spin-lock (CESL) magnetic resonance imaging (MRI). Labeling in the case of CEST MRI works non-invasively by selective radiofrequency (rf) irradiation: e.g. hydroxyl protons of glucose are labeled by means of rf irradiation that matches their chemical shift and their proton exchange regime. This labeling is transferred to water protons by chemical exchange and can be detected via MRI. The feasibility to track the uptake of glucose in animals was proven employing both techniques, CEST^1^,^2^,^3^,^4^,^5^ and CESL^6^,^7^,^8^. First results in human tumor patients were recently published by Xu *et al*. and Wang *et al*. by means of CEST^9^,^10^ and by Schuenke *et al*. employing an adiabatically prepared CESL technique^11^.

However, the history of many MRI contrasts showed that the translation of new contrasts into clinical routine requires a fast and robust technique and an evaluation process, which is as simple as possible. To fulfill those clinical needs, we show herein that CESL-based dynamic glucose enhanced MRI (DGE-MRI) can be accelerated essentially and made robust against field inhomogeneities by means of adiabatically prepared T_1ρ_-weighted (T_1ρ_-w) imaging. Further, we introduce a simple but appropriate evaluation method that provides a quantitative T_1ρ_-w DGE contrast. In simulations and *in vitro* experiments, we demonstrate that the proposed contrast depends linearly on glucose concentration changes and is independent of tissue-specific relaxation parameters. After implementation and optimization at a 7T MRI scanner, this technique was employed in a glucose-injection experiment with seven-second temporal resolution. First results of T_1ρ_-w DGE-MRI in a patient with glioblastoma are presented revealing a substantial DGE contrast between tumor and healthy tissue.

As the origin of the DGE contrast is still under discussion^1^,^2^,^3^,^4^,^5^,^6^,^7^,^8^,^9^,^10^,^11^, the presented technique does not only form a simple and robust diagnostic tool for studying the DGE contrast in clinical studies, but with its high temporal resolution also serves as a research tool. Thus, it might help solving the question to what extent the occurring contrast originates from intra- or extracellular glucose level changes, from pH changes, or from other glucose related metabolites.

## Results

### T_1ρ_-weighted DGE-MRI *in vivo*

**Figure 1:**
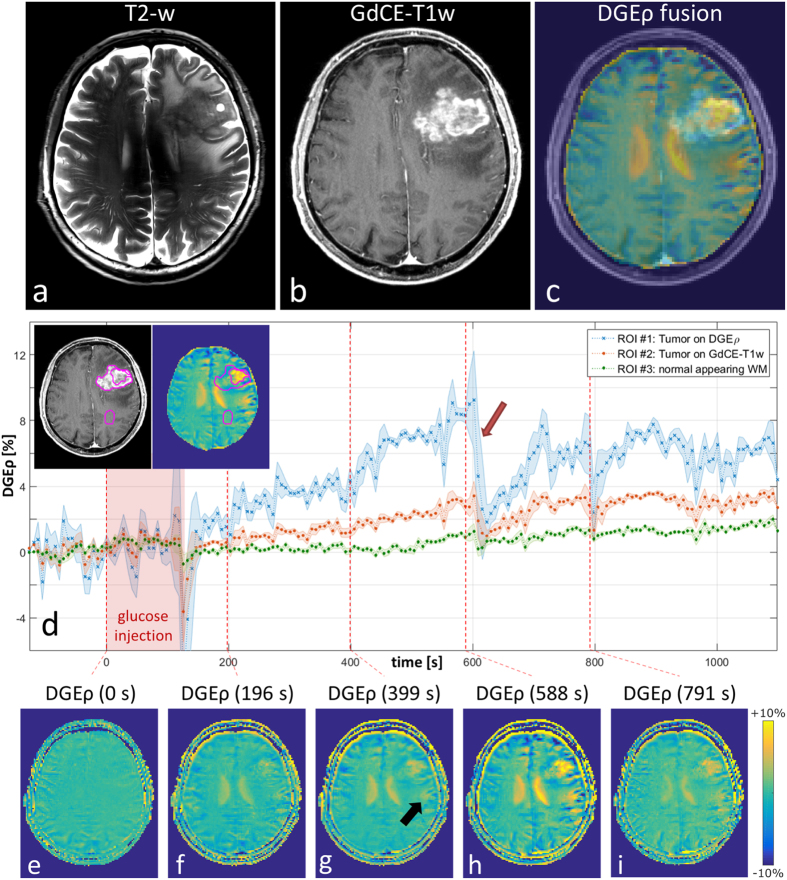
Accelerated and quantitative T_1ρ_-weighted dynamic glucose enhanced MRI applied in the study of a patient with a glioblastoma at B_0_ = 7T. (**a**) T_2_-weighted image acquired at 7T, (**b**) gadolinium-enhanced T_1_-weighted (GdCE-T1w) image acquired at 3T, (**c**) fusion of the GdCE-T1w image and the T_1ρ_-weighted dynamic glucose enhancement (DGE_ρ_) obtained at t = 588 s. (**d**) Unsmoothed DGE_ρ_ time curves with a temporal resolution of less than 7 seconds in a tumor-ROI selected on DGE_ρ_ (ROI #1), a second tumor-ROI selected on the GdCE-T1w image (ROI #2), and a ROI in normal appearing white matter (ROI #3). The error is given by the standard deviation of 5 consecutive data points and the ROIs are marked in the GdCE-T1w and DGE_ρ_ image shown in the top left corner. Increasing DGE_ρ_ values are obtained in both tumor-ROIs after the end of the glucose injection. The red arrow marks an abrupt signal drop induced by patient motion. (**e**–**i**) DGE_ρ_ images (average of 5 consecutive images) at different time points after glucose injection. Note the hyperintense region at the bottom of the tumor area (black arrow; (**g**)), which is not visible in the GdCE-T1w image (**b**).

The accelerated and quantitative T_1ρ_-w DGE-MRI protocol optimized with respect to contrast and scanning time was applied with temporal resolution of less than 7 seconds in the study of a patient with a brain tumor. The tumor (glioblastoma, WHO grade IV) located in the left frontal lobe can be identified in the T_2_-w image acquired at 7T (Fig. 1a) and in the co-registered gadolinium contrast-enhanced T_1_-weighted (GdCE-T1w) image obtained at 3T (Fig. 1b). We define the quantitative T_1ρ_-weighted dynamic glucose enhancement (DGE_ρ_) by the relative signal difference (Eq. 4 in Methods) at each time point


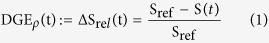


for evaluation of series of T_1ρ_-w *in vivo* images. DGE_ρ_ depends linearly on glucose concentration changes and is independent of tissue-specific relaxation parameters as demonstrated in simulations and *in vitro* experiments (see below). DGE_ρ_ was calculated for each time point in every voxel employing the average of 18 T_1ρ_-w images acquired before start of the glucose injection as reference (S_ref_). First of all, the DGE_ρ_ images obtained after glucose injection (cf. Fig. 1c, t = 588 s) clearly delineate the tumor region consistent with the GdCE-T1w image (Fig. 1b). We further evaluated DGE_ρ_ as a function of time in three regions of interest (ROIs), namely a tumor-ROI (ROI #1) selected on the DGE_ρ_ image shown in Fig. 1c, a second tumor-ROI (ROI #2) selected on the GdCE-T1w image (Fig. 1b), and a ROI in normal appearing white matter (ROI #3). The ROI-specific DGE_ρ_ curves are shown in Fig. 1d. The ROIs are marked in the GdCE-T1w and DGE_ρ_ image shown in the top left corner. Before the start of glucose injection (at t = 0 s) DGE_ρ_ of all three ROIs fluctuated around 0% and consequently no tumor contrast was visible in the corresponding DGE_ρ_ image shown in Fig. 1e. After the start of injection, all curves slightly increased and a faint contrast became apparent in the tumor area as well as in the paraventricular area about 1 min after the end of the injection phase (Fig. 1f). For t ≥ 200 s the curves of both tumor ROIs were outside the error of normal appearing white matter (ROI #3), which showed only a minor increase in the DGE_ρ_ curve over the entire time course. Accordingly, the contrast in the DGE_ρ_ images increased, revealing another slightly enhancing region (black arrow) at the bottom of the tumor area (Fig. 1g), which remained visible in the DGE_ρ_ images obtained afterwards (Fig. 1h and i). The highest contrast was observed at about 10 min after start of the injection (Fig. 1h), where the T_1ρ_-w dynamic glucose contrast in ROI #1 was more than twice as high compared with that in ROI #2 and about 8 times higher than in normal appearing white matter. The subsequent signal drop in the DGE_ρ_ curve (red arrow in Fig. 1d) was most likely due to patient motion, which was identified by a displacement of the brain position in the time-resolved T_1ρ_-w images. Interestingly, the DGE_ρ_ images did not show any contrast in blood vessels.

### Bloch-McConnell simulations

**Figure 2:**
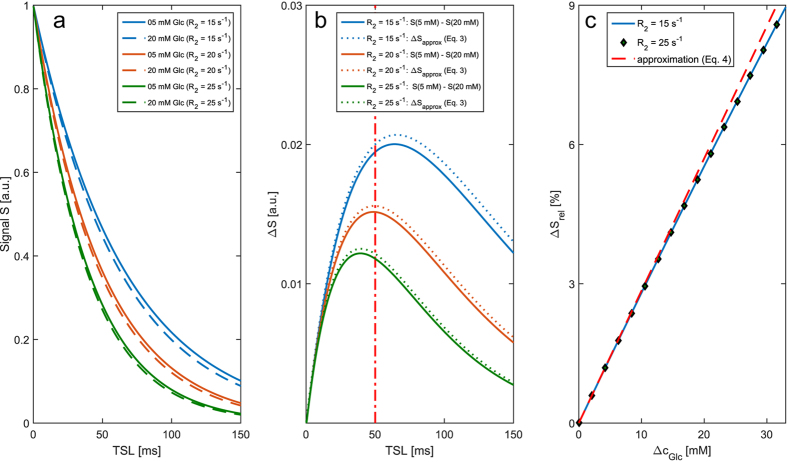
Simulation of T_1ρ_ relaxation curves and investigation of T_1ρ_-weighted glucose contrast. (**a**) Simulated T_1ρ_ relaxation curves for glucose concentrations of 5 mM and 20 mM and transversal relaxation rates R_2_ = 15 s^−1^, 20 s^−1^ and 25 s^−1^. (**b**) Signal difference ΔS between the relaxation curves (solid lines) and our analytical approximation (Eq. 3, dotted lines) as a function of the spin-lock time TSL. The dashed vertical line at TSL = 50 ms marks the suggested value yielding the best contrast-to-noise ratio. (**c**) The proposed contrast ΔS_rel_ as function of the glucose concentration change Δc_Glc_ for R_2_ = 15 s^−1^ (solid blue line) and R_2_ = 25 s^−1^ (green diamonds) for constant TSL = 50 ms. Note the linearity in Δc_Glc_ and the independence of absolute relaxation rates.

To investigate the contrast obtained with T_1ρ_-w MRI we simulated T_1ρ_ relaxation curves by means of a Bloch-McConnell simulation tool. Figure 2a shows the relaxation curves for glucose concentrations of 5 mM and 20 mM and transversal relaxations rates R_2_ = 15 s^−1^, 20 s^−1^ and 25 s^−1^. Figure 2b displays the difference ΔS between the simulations for 5 mM and 20 mM for the three R_2_ (solid lines) together with analytical approximations (dashed lines) obtained with equation (3) (see in Methods below). The approximation agrees well; especially the maxima appear at the same position proving its validity. As predicted by equation (3) the curves differ for varying R_2_ making ΔS an inappropriate measure for changes of glucose concentration *in vivo*. However, the approximation suggests that this dependency on R_2_ can be eliminated by dividing equation (3) by the reference signal 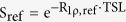. This yields the relative signal change ΔS_rel_ given by equation (4), that also defines DGE_ρ_(t) (Eq. 1) at a specific time point t. ΔS_rel_, and thus DGE_ρ_(t) depends only on TSL and the variation ΔR_ex_ of the exchange-dependent relaxation rate R_ex_. In Fig. 2c ΔS_rel_ is plotted as a function of the glucose concentration change Δc_Glc_ for R_2_ = 15 s^−1^ (solid blue line) and R_2_ = 25 s^−1^ (green diamonds) for one specific spin-lock time of 50 milliseconds. The plot shows that the contrast does not depend on R_2_. The analytical approximation (Eq. 4; dashed red line) agrees well again. Thus, DGE_ρ_(t) defined by ΔS_rel_ represents a quantitative contrast, which depends linearly on ΔR_ex_ and hence on changes of the glucose concentration (Δc_Glc_) for a given TSL. To determine the optimum TSL (TSL_opt_) one has to consider not only the signal-to-noise ratio (SNR), but also the contrast-to-noise ratio (CNR). Assuming a constant SNR, the maximum CNR is given by the position of the maxima of ΔS. Equation (3) allows to determine this point analytically yielding TSL_opt_ =T_1ρ_. For the simulated relaxation rates, which represent the range we observed in human brain tissue at 7T using the adiabatically prepared spin-lock approach^11^, the CNR for TSL = 50 ms (red vertical line in Fig. 2b) is close to the optimal value for all relaxation rates that we considered.

### *In vitro* experiments

**Figure 3:**
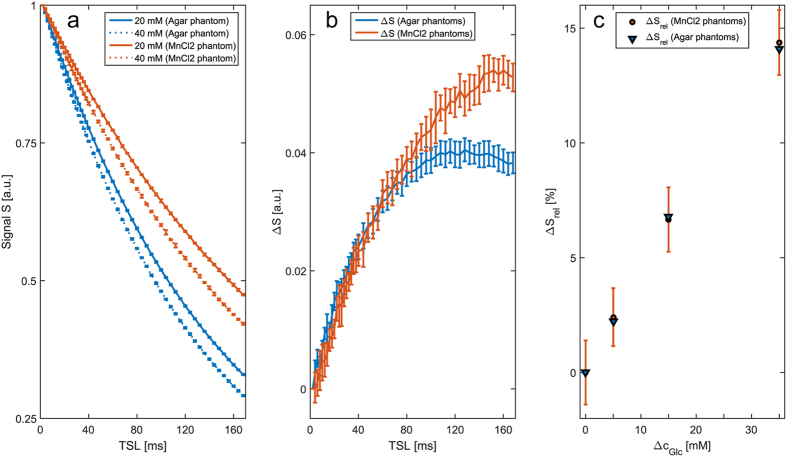
Measurements of aqueous solutions with different glucose concentrations and different R_1_ and R_2_ to confirm the proposed contrasts’ independence of absolute relaxation rates and linearity in the glucose concentration. (**a**) Measured T_1ρ_ relaxation curves for glucose concentrations of 20 mM and 40 mM and different relaxation rates adjusted using Agar and gadoteric acid (“Agar phantoms”) and Manganese dichloride (“MnCl2 phantoms”). (**b**) Signal difference ΔS between the measured relaxation curves for 20 mM and 40 mM as a function of TSL for the Agar and MnCl2 phantoms, respectively. (**c**) ΔS_rel_ obtained for constant TSL = 100 ms as function of the glucose concentration change Δc_Glc_ for the Agar and MnCl2 phantoms, respectively. The consistency of both curves is in agreement with our simulations (cf. Fig. 2c) and proves the independence of the contrast on absolute relaxations rates.

To confirm the results of our simulations we performed measurements of aqueous solutions with different glucose concentrations and different R_1_ and R_2_. The relaxation rates were adapted using gadoteric acid and agar for one set of solutions and MnCl_2_ for a second set. In the following, the different sets are called agar phantoms and MnCl_2_ phantoms, respectively. The measured T_1ρ_ relaxation curves for glucose concentrations of 20 mM (solid lines) and 40 mM (dashed lines) are plotted in Fig. 3a for the agar and the MnCl_2_ phantoms. The curves were normalized to the first value and all data points represent the mean and standard deviation of three independent measurements. Figure 3b shows the signal differences ΔS between the two particular relaxation curves from Fig. 3a. The curves display the dependence on the absolute relaxation rates expected from the simulations (Fig. 2b) and equation (3). Figure 3c shows the relative signal difference ΔS_rel_ for a constant spin-lock time of 100 milliseconds as a function of the glucose concentration change. The curves for both, the agar and the MnCl_2_ phantoms, agree within the errors (shown only for the MnCl_2_ phantom measurements for the sake of clarity). The consistency of both curves proves the independence of the relative signal difference ΔS_rel_ (or rather DGE_ρ_ for series of *in vivo* images) on absolute relaxations rates.

## Discussion

In this study, we showed that T_1p_-based DGE-MRI can be accelerated essentially by employing T_1ρ_-w imaging. The introduced contrast called T_1ρ_-w dynamic glucose enhancement (DGE_ρ_, Eq. 1) was shown to be independent of relaxation parameters of tissue and direct proportional to changes of the glucose concentration thus enabling fast and quantitative DGE-MRI in a glioblastoma patient with a temporal resolution of less than 7 seconds.

So far glucose enhanced MRI in humans has been performed in brain tumor patients at 7T by means of CEST^9^ and T_1ρ_ mapping^11^ and in head and neck tumor patients at 3T by means of CEST^10^. In all studies, an increased glucose uptake was reported after intravenous injection of natural D-glucose. However, the studies substantially differed in the temporal resolution, varying between 5 seconds in the case of CEST-based dynamic glucose enhanced MRI applied by Xu *et al*.^9^ and about 5 minutes in the study of Wang *et al*.^10^. The temporal resolution of the T_1p_-weighted approach proposed in this study is below seven seconds and thus in the same order as for CEST-based DGE-MRI. As the spin-lock preparation time of 50 ms is much shorter compared to CEST saturation, which normally requires seconds, the temporal resolution can be increased to about 3 s if SNR is sufficient.

High temporal resolution is mandatory to detect variations on small time scales like changes in the blood glucose level (BGL) after a bolus glucose injection. Robust tracking of the BGL could potentially enable pharmacokinetic modelling based on compartment models as for example employed in gadolinium-based dynamic contrast enhanced MRI (DCE-MRI)^12^. Another benefit of a high temporal resolution is the opportunity to increase the effective SNR and CNR by averaging of several consecutive measurements. This could be relevant for glucose enhanced MRI when a lower temporal resolution is sufficient, e.g. when the bolus injection is replaced by a continuous glucose infusion, but also for native T_1ρ_-based imaging without glucose enhancement. Consequently the presented adiabatically prepared T_1ρ_-w imaging technique with the proposed normalization might also improve cartilage imaging, where T_1ρ_ mapping is a common technique to detect the loss of proteoglycan in the early stages of osteoarthritis^13^,^14^,^15^,^16^.

As shown previously, an adiabatically prepared spin-lock approach combined with a non-adiabatic MRI readout, as used in our study, works within specific absorption rate (SAR) restrictions and technical limitations of ultrahigh field whole-body scanners^11^. This leads to a homogenous T_1ρ_ contrast over the entire brain despite B_1_ inhomogeneities and consequently to negligible contributions from B_1_ dispersion to the DGE contrast^11^. We want to point out that for the *in vivo* T_1ρ_-w DGE-MRI measurement, SAR was around 50% of the allowed value and hence relatively low for using adiabatic pulses. This can be understood since only two adiabatic half-passage pulses are used per 7 s. Consequently, a reduction of the recovery time and thus an increase of the temporal resolution is also in accordance with SAR restrictions. Furthermore, the proposed T_1ρ_-w DGE-MRI inherits all benefits of the adiabatically prepared spin-lock approach. This includes the higher sensitivity to the intermediate and fast exchange regime relevant for glucose and the enhanced robustness against B_0_ inhomogeneities compared to CEST^6^,^17^, but also the fact that changes in DGE_ρ_ due to inhomogeneities in the B_1_ field are negligible compared to changes induced by variations of the glucose concentration^11^. Especially the robustness against field inhomogeneities qualifies the presented approach for application at whole-body ultra-high field scanners. These are of great interest for chemical exchange sensitive experiments due to the increasing exchange-weighting with higher field strength^18^,^19^. Further, the robustness against field inhomogeneities makes the application of correction methods dispensable and thus simplifies the post-processing.

As predicted by our analytical approximation (Eq. 3) we could show that the dependency of the signal difference (ΔS) on absolute relaxation rates can be eliminated by an appropriate normalization yielding the T_1ρ_-weighted dynamic glucose enhancement (DGE_ρ_), which depends linearly on the glucose concentration and is independent of relaxation parameters of the tissue. These properties could be verified with simulations (Fig. 2c) and *in vitro* measurements (Fig. 3c). We want to point out that the intrinsic robustness of the adiabatic spin-lock against field inhomogeneities in combination with the introduced normalization yield a quantitative contrast, which can be compared between different measurements and subjects. CEST-based DGE-MRI techniques, on the other hand, can be prone to influences of inhomogeneities and absolute relaxation rates^20^. Although faster T_2_ relaxation due to exchange^21^ can lead to a signal enhancement in CEST-based DGE-MRI, being independent of T_1_ and T_2_ relaxation has the benefit of the above mentioned quantitative contrast and additionally some practical benefits: with DGE_ρ_ it is possible to perform a DGE measurement after gadolinium injection which is practical in clinical routine. Beyond that, it is also thinkable to perform DGE and DCE with the same injection bolus at the same time, which would speed up the acquisition and provides a reference for pharmacokinetic investigations.

**Figure 4:**
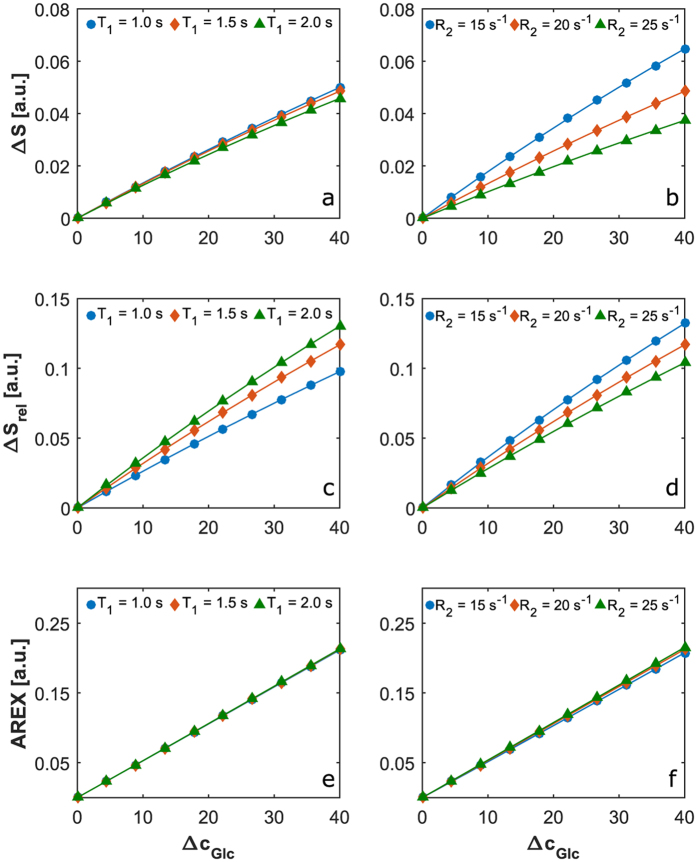
Comparison of different metrics for CEST-based DGE-MRI by means of Bloch-McConnell simulations. The metric ΔS = (S_ref_ − S)/S_0_ and, contrary to T_1ρ_-w DGE-MRI, also the metric ΔS_rel_ = (S_ref_ − S)/S_ref_ show a dependency on relaxation times T_1_ and T_2_ in the case of CEST-based DGE-MRI (**a**–**d**). However, employing R_1_ = 1/T_1_ and the apparent exchange-dependent relaxation evaluation AREX = (S_0_/S_ref_ − S_0_/S) · R_1_ also CEST-based DGE-MRI yields a relaxation independent contrast. The simulated CEST pre-saturation parameters were chosen similar to Xu *et al*.^9^: 32 sinc-gauss pulses (50 ms, Δω = 1.2 ppm, B_1_ = 1.96 μT, separated by a 25 ms delay, each) and a delay of 2 s after each scan. The water and solute pool parameters were chosen similar to the CESL simulations in Fig. 2.

However, also in the case of CEST the influences of absolute relaxation rates can be handled by employing relaxation compensation techniques. Figure 4 shows simulated T_2_- and T_1_-dependencies of the CEST-based DGE approach based on the paper of Xu *et al*.^5^. Our simulations reveal that by using a T_1_ map and the AREX^22^ metric, also quantitative CEST-based DGE-MRI can be realized.

### DGE_ρ_ in brain tumor patient

Applying the proposed quantitative T_1ρ_-w DGE-MRI approach with seven-second temporal resolution evaluated using DGE_ρ_ in a glioblastoma patient we observed an increasing DGE contrast in the tumor area after the intravenous glucose bolus injection. This finding is in agreement with the outcome of our previous DGE-MRI study of a glioma patient based on T_1ρ_ mapping^11^ and the *in vivo* study of Xu *et al*.^9^ employing CEST-based DGE-MRI in brain tumor patients. A quantitative evaluation of DGE_ρ_ in three regions of interest (ROIs) revealed a substantially increased contrast in the tumor ROIs selected on the DGE_ρ_ and GdCE-T1w images compared to normal appearing white matter. Interestingly, the hyperintense tumor areas in the DGE_ρ_ images (cf. Fig. 1f–i) partially overlap but still differ from those on the GdCE-T1w image (Fig. 1b). The observed difference in both contrasts is in agreement with the findings of Walker-Samuel *et al*.^3^, who did not observe a significant correlation between glucoCEST and GdCE-T1w contrast in an animal study. This allows for the conclusion that DGE-MRI can provide complementary information about pathologies compared to contrast enhanced T1-w MRI, which is the current gold standard method for detecting and characterizing high-grade glioma^23^ by visualizing blood brain barrier (BBB) disruption. We could not validate whether the enhancing region outside the tumor area (black arrow; Fig. 1g), which was not visible in the native T_2_-w and GdCE-T1w images (Fig. 1a and b) was an active tumor region or not. Hence, it remains to be shown if DGE-MRI might highlight hidden active regions of the tumor and thus forms a tool for the early detection of cancer. Whereas CEST-based DGE-MRI showed an uptake in blood vessels^9^, this was not observed by T_1p_-w DGE-MRI. It remains to be investigated in detail if this is due to the short saturation period of spin-lock compared to CEST or if it has a meaning on the contrast origin level.

### Origin of DGE_ρ_ contrast

We showed that the DGE_p_ signal increases in tumors. However, the actual origin of the signal changes in DGE-MRI is still under discussion^1^,^2^,^3^,^4^,^5^,^6^,^7^,^8^,^9^,^10^,^11^. Chan *et al*.^1^ stated that the signal in glucoCEST originates mostly from the extracellular compartment, and, due to lower pH, predominantly from the extracellular-extravascular glucose. Further, Chan *et al*.^1^ as well as Walker-Samuel *et al*.^3^ showed that FDG-PET and glucoCEST MRI are enhancing similarly. In contrast to Chan *et al*., Walker-Samuel *et al*. concluded from the similarity with FDG-PET that also intracellular compounds contribute to the glucoCEST signal. This conclusion was also based on their results of ^13^C spectroscopy after injection of ^13^C labeled glucose that showed appearance of glucose, glucose-6-phsophate, fructose phosphates, as well as amino acids such as glutamate, glutamine, taurine and alanine. From phantom experiments they further conclude that glucose and its metabolic products as well as glutamate and glutamine might contribute to the glucoCEST signal, but lactate protons are exchanging too fast to be detectable with CEST^3^. For the case of glucoCESL, Jin *et al*.^6^ also mention the contribution of glucose metabolism products. Thus, it is still under discussion to what extent DGE-MRI is extracellular and consequently only with indirect access to metabolism, or intracellular, which would give more insight to metabolism. From our data, we can only conclude that changes in T_1ρ_-based DGE-MRI originate from a different compartment than in gadolinium enhanced MRI, which is coherent with both origins, the extracellular extravascular and the intracellular space or a mixture of both. This conclusion is also in coherence with previous publications^1^,^3^,^5^,^11^. However, in accordance with Jin *et al*.^6^ we want to point out that with on-resonant T_1ρ_-based DGE-MRI all exchanging sites contribute to the signal and, compared to CEST, also the close to water resonating and faster exchanging pools such as lactate have a stronger contribution, as sensitivity of spin-lock to high exchange rates is improved^6^,^17^. As the presented technique can track the signal changes fast and accurate, it might become an important tool for further investigations of the origin of the DGE contrast.

Having shown that our contrast is quantitative, we can employ the *in vitro* calibration to try calculating the corresponding glucose concentration *in vivo* similar to Jin *et al*.^6^. Assuming the relaxivity measured in phantoms (Fig. 3) to be valid also *in vivo*, the obtained DGE_ρ_ or rather change of R_1ρ_ in the tumor would correspond to a glucose concentration increase of up to 40 mM (721 mg/dL), using the relaxivity reported by Jin *et al*.^6^ the concentration change would be approximately 25 mM (450 mg/dL). Although Xu *et al*. measured a venous glucose level of up to 23.7 mM (427 mg/dL) in volunteers about 2–4 min after the injection^9^, a value between 25 mM and 40 mM still seems to be improbably high. This hints that the observed signal change might not solely originate from the hydroxyl exchange of glucose, but as discussed above, also from glucose metabolic products^3^,^6^, and glutamate and glutamine^3^. Moreover, the relaxivity of the DGE effect potentially differs between the *in vivo* and *in vitro* situation as it depends on temperature, pH, and the concentration of exchange catalysts and has not yet been determined directly *in vivo* or even in tumors.

### Unexpected signals and motion correction

After injection, glucose is also expected to rapidly enter the cerebrospinal fluid (CSF) leading to an increase of R_1ρ_ and consequently to a positive DGE contrast in the ventricles as observed in our measurements. However, it has also been reported that a glucose injection results it volumetric changes of the CSF compartments^24^. This is an explanation for the negative contrast observed in the ventricles by Xu *et al*. employing CEST-based DGE-MRI^9^. Such volumetric changes can also lead to a reduction of R_1ρ_ and consequently to a negative DGE contrast employing T_1ρ_-weighted DGE-MRI explaining the observed signals in the outer CSF compartments, where pixels are expected to be affected by partial volume effects, which most likely result from the limited special resolution in z-direction. Volumetric changes of the CSF lead to an increase of the CSF fraction in the partial volume affected voxels and consequently to negative DGE contrasts, as R_1ρ_ of CSF is about one order of magnitude smaller than for brain tissue^8^,^11^. This insight must be included when interpreting DGE uptake of tumors close to CSF regions.

Generally, patient motion is a problem of every contrast based on signal differences between different time points, including all CEST- and CESL-based DGE-MRI approaches, but also dynamic contrast enhanced (DCE) MRI or functional MRI (fMRI). For correction of motion after data acquisition, we employed a rigid registration algorithm. However, for a robust post-process correction of extensive out-of-plane motion the acquisition of an expanded volume is mandatory; for example by applying single-shot 3D MRI sequences such as 3D gradient echo-based MRI^25^ or echo-planar imaging (EPI) speed-up^26^, which can easily be combined with the T_1ρ_-weighted preparation^11^. An alternative method to reduce patient motion is the application of immobilization devices known from radiation therapy as done by Wang *et al*.^10^. In principle, also a combination of post-process motion correction and immobilization of the patient is possible.

In conclusion, dynamic glucose enhanced MRI (DGE-MRI) might open the window to non-invasive observation of glucose uptake and potentially metabolism. Due to its high temporal resolution in combination with a high robustness against field inhomogeneities and a high sensitivity to glucose, T_1ρ_-weighted DGE-MRI has a high potential to facilitate the translation of glucose enhanced MRI into the clinics. The simple quantitative evaluation can be performed online directly at the scanner to fulfill the clinical demand for practicability. Quantitative DGE further allows a deeper insight into the underlying correlations and in principle enables combined measurements with relaxation affecting contrast agents such as Gd. Further longitudinal studies with larger numbers of patients with different tumor grades are planned to investigate the full potential for detection and staging of cancer or also neurodegenerative diseases by means of the proposed fast and quantitative T_1ρ_-w DGE-MRI technique.

## Methods

### R_1ρ_ theory and glucose contrast

For a two-pool system (one water, one solute proton pool) the on-resonant longitudinal relaxation rate in the rotating frame R_1ρ_ is given by^27^ R_1ρ_ = R_2_ + R_ex_ where R_2_ is the transverse relaxation rate of water protons without contributions from chemical exchange and R_ex_ the exchange dependent relaxation rate. R_ex_ can be approximated as^6^


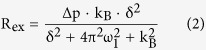


where Δp is the ratio of concentrations of solute and water protons, k_B_ is the exchange rate (units of s^−1^) and δ the resonance shift (units of rad/s) between the solute and water proton pools, and ω_1_ = γB_1_ is the amplitude of the spin-lock pulse (units of rad/s). For T_1ρ_-weighted MRI we could show that the difference in signal intensities (ΔS) between a voxel and a reference voxel with different exchange-dependent relaxation, e.g. gray and white brain matter, can be approximated as^11^


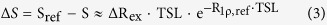


assuming that ΔR_ex_ · TSL ≪ 1, where ΔR_ex_ is the difference of the exchange dependent relaxation rates between the two voxels and TSL is the spin-lock time. This formula also holds for the same voxel but different time points, e.g. in T_1ρ_-w DGE-MRI before and after administration of glucose. A similar metric was used by Xu *et al*. for the evaluation of CEST-based DGE-MRI data^5^,^9^.

The dependence of ΔS on R_1p_ and hence on R_2_ (Eq. 3) indicates that ΔS might be a non-optimal measure for glucose concentration changes *in vivo* since R_2_ varies between different tissue types. Dividing equation (3) by the reference signal 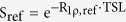 yields the potentially more robust relative signal change:


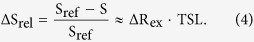


### Simulations and *in vitro* measurements

For simulations the Bloch-McConnell equations^28^ for two pools, one bulk water and one solute pool, were solved numerically as described in Zaiss and Bachert^19^ using MATLAB (MATLAB R2015b, 2015; The MathWorks Inc., Natick, Massachusetts, USA). The simulation parameters were: R_1_ = 0.66 s^−1^, R_2_ = 20 s^−1^, δ = 1.5 ppm, k_B_ = 3 kHz, Δp = 9.0·10^−4^ (≙20 mM), B_1_ = 5 μT and TSL = 50 ms. For the *in vitro* measurements two sets of phosphate buffered aqueous solutions (pH ≈ 7.2) with glucose concentrations of 5 mM, 10 mM, 20 mM and 40 mM were used. The relaxation times of the solutions were adjusted by means of 0.095 mM gadoteric acid (Dotarem®; Guerbet, France) and 1.6% agar for the first set of solutions (“agar phantoms”) and 0.45 mM MnCl_2_ for the second set (“MnCl_2_ phantoms”).

### Patient examination

**Figure 5:**
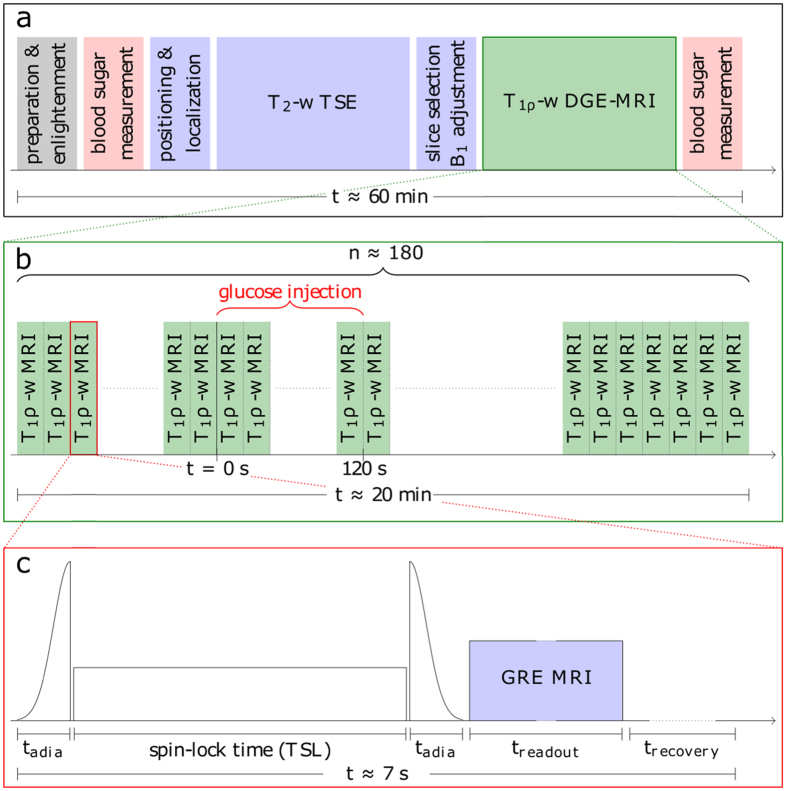
Schema of the patient examination protocol at 7T (**a**) including the T_1ρ_-w DGE-MRI part (**b**). The DGE-MRI part consists of 178 T_1ρ_-w image acquisition of which 18 were performed before the start of glucose injection at t = 0 s. (**c**) Detailed schema of an individual T_1ρ_-w acquisition consisting of an adiabatic spin-lock preparation, a conventional gradient echo MRI readout and a recovery time. A single T_1ρ_-w acquisition took about 7 seconds resulting from 66 ms spin-lock preparation time (TSL = 50 ms, t_adia_ = 8 ms), an MRI readout time of about 2.5 s and a recovery time of 4 s.

As part of a clinical study T_1ρ_-w DGE-MRI was applied in the examination of a 66-year-old male patient with newly diagnosed and histopathologically confirmed glioblastoma (WHO grade IV). The examination was approved by the local ethics committee of the Medical Faculty of the University of Heidelberg and is in accordance with the relevant guidelines and regulations. Written informed consent was received from the patient prior to the examination. The patient was examined after a 6-hour fasting period ensuring a normal blood glucose level before injection. Using an intravenous line 100 ml of 20% *D*-glucose (SERAG-WIESSNER GmbH & Co. KG, Naila, Germany) were injected manually over 2 min into an arm vein. Two blood samples were taken, one before and the other approximately 25 min after the glucose injection. The blood sugar values, determined by means of a conventional blood sugar meter (Accu-Chek Aviva; Roche Diagnostics, Rotkreuz, Switzerland), were 106 mg/dL (5.9 mM) and 146 mg/dL (8.1 mM) pre- and post-injection, respectively. The complete protocol of the patient examination is sketched in Fig. 5a.

The total examination time, including patient preparation and positioning, morphological and T_1ρ_-w DGE MRI, as well as the blood glucose measurements, was approximately 60 min. The T_1ρ_-w DGE-MRI part shown in Fig. 5b consisted of n = 178 individual measurements leading to an acquisition time of about 20 minutes. The first 18 measurements were performed before the start of the glucose injection and yielded the reference for the calculation of the dynamic glucose enhancement (Eq. 1).

### Data acquisition and analysis

All MR measurements were performed on a 7T whole-body MR scanner (MAGNETOM 7T, Siemens Healthcare, Erlangen, Germany) using a 24-channel head coil (Nova Medical, Wilmington, MA, USA). The MR sequence used for T_1ρ_-based MRI consists of an adiabatically prepared spin-lock pulse cluster as described in Schuenke *et al*.^11^ and shown in Fig. 5c followed by a conventional MRI readout. The parameters of the adiabatic hypsec-pulses were: RF amplitude B_1, max_ ≥ 20 μT, adiabatic sweep time t_adia_ = 8 ms, bandwidth Δ = 1200 Hz, and μ = 6, where μ is a dimensionless parameter that controls the pulse shape^29^. The spin-lock frequency was adjusted manually to obtain the desired value of B_1_ ≈ 5 μT in the region of interest, e.g. the tumor area. For MRI readout we used a centric-reordered single-shot gradient echo (GRE) sequence. *In vivo* we acquired three axial slices in an interleaved way (matrix = 128 × 104, FoV = 220 × 178 mm^2^, T_E_ = 3.61 ms, T_R_ = 23 ms, flip angle = 10°, slice thickness = 5 mm, distance factor = 20%). The same MR sequence with an adapted preparation block was used to obtain B_1_ maps by means of the WASABI^30^ approach.

In the patient examination we further acquired a stack of 32 high-resolution (0.4 × 0.4 × 2 mm^3^) T_2_-weighted images using a Turbo-Spin-Echo (TSE) sequence (T_E_ = 52 ms, T_R_ = 12340 ms). The Gadolinium contrast-enhanced T_1_-weighted (GdCE-T1w) images were acquired 10 days prior to the 7T examination in the course of a clinical MR protocol at 3T. The GdCE-T1w, T_2_-w and T_1ρ_-w images were co-registered and the slice thickness of the GdCE-T1w and T_2_-w images was interpolated to the slice thickness (5 mm) of the T_1ρ_-w images using a multi modal rigid registration algorithm in the DKFZ Image Processing Platform - an in-house version of the Medical Imaging Interaction Toolkit (MITK)^31^. All further post-processing and data analysis, including a rigid in-plane motion correction of the T_1ρ_-w images was performed using self-written software in MATLAB. All errors were calculated taking into account the law of error propagation.

## Additional Information

**How to cite this article**: Schuenke, P. *et al*. Fast and Quantitative T1ρ-weighted Dynamic Glucose Enhanced MRI. *Sci. Rep.* **7**, 42093; doi: 10.1038/srep42093 (2017).

**Publisher's note:** Springer Nature remains neutral with regard to jurisdictional claims in published maps and institutional affiliations.
